# Advancing Duodenoscope Reprocessing with Alginate-Coated Calcium Peroxide Nanoparticles

**DOI:** 10.3390/life15111643

**Published:** 2025-10-22

**Authors:** Adrian Fifere, Cristian-Dragos Varganici, Elena-Laura Ursu, Tudor Pinteala, Vasile Sandru, Ioana-Andreea Turin-Moleavin, Irina Rosca, Gheorghe G. Balan

**Affiliations:** 1 Petru Poni Institute of Macromolecular Chemistry, Grigore Ghica Voda Alley 41A, 700487 Iasi, Romania; fifere@icmpp.ro (A.F.); varganici.cristian@icmpp.ro (C.-D.V.); ursu.laura@icmpp.ro (E.-L.U.); tudor_pinteala@umfiasi.ro (T.P.); 2 Faculty of Medicine, “Grigore T. Popa” University of Medicine and Pharmacy, 700115 Iasi, Romania; balan.gheo@me.com; 3 Department of Gastroenterology, Carol Davila University of Medicine and Pharmacy, 050474 Bucharest, Romania; drsandruvasile@gmail.com; 4 Clinical Emergency Hospital of Bucharest, 105402 Bucharest, Romania; 5 St. Spiridon Iasi County Emergency Clinical Hospital, 700111 Iasi, Romania

**Keywords:** duodenoscope reprocessing, calcium peroxide nanoparticles, alginate, disinfectant, antibacterial

## Abstract

Background/Objectives: Although significant advances in duodenoscope reprocessing have been introduced since mid-2010s—including enhanced cleaning protocols, disposable distal endcaps, and the introduction of fully single-use duodenoscopes—residual contamination and infection risks remain unresolved. Moreover, repeated reprocessing may cause cumulative damage to the polymer surfaces, elevator mechanisms, and internal channels of the duodenoscopes, making them more susceptible to residual contamination. To minimize the duodenoscope polymer degradation caused by intensive use and reprocessing, new alternatives are urgently needed. In this context, calcium peroxide nanoparticles coated with sodium alginate (CaO_2_–Alg NPs), synthesized by our group, were tested for the first time as a disinfectant capable of combating nosocomial pathogens while reducing device deterioration associated with repeated investigations and reprocessing. Methods: The disinfectant properties of the CaO_2_–Alg NPs were evaluated under biomimetic conditions using reference bacterial strains commonly associated with nosocomial infections. In addition, the compatibility of the nanoparticles with the polymeric duodenoscope coatings was assessed after simulated intensive use. The external polymer coating was structurally and morphologically characterized by Fourier Transform Infrared Spectroscopy (FTIR), Differential Scanning Calorimetry (DSC), Atomic Force Microscopy (AFM), and Scanning Electron Microscopy (SEM). Results: The nanoparticles exhibited important antimicrobial activity against the reference bacterial strains *Staphylococcus aureus*, *Escherichia coli*, *Enterococcus faecalis*, and *Klebsiella pneumoniae* after only 20 min of incubation. Intensive exposure to the CaO_2_–Alg NPs did not cause additional structural or morphological damage to the duodenoscope’s external polymers and did not alter their anti-adhesive properties. Conclusions: The CaO_2_–Alg NPs appear to be a safe and effective disinfectant for the duodenoscope reprocessing, offering both antimicrobial efficacy and material compatibility.

## 1. Introduction

Duodenoscope-associated infections remain a critical patient-safety concern in 2025. Despite nearly a decade of intensified reprocessing protocols, mandatory US Food and Drug Administration (FDA) post-market surveillance, and the introduction of innovative technologies such as disposable distal endcaps and fully single-use duodenoscopes, the problem has not been eradicated [1]. Contamination rates of up to 15% have been documented in so-called “patient-ready” duodenoscopes [2], and even under enhanced surveillance methods, 5–9% still harbour high-concern pathogens [3,4]. While large-scale outbreaks have become less frequent, any transmission of multidrug-resistant organisms carries profound clinical and public health consequences, in line with Centers for Disease Control and Prevention (CDC) alerts on Carbapenem-resistant Enterobacteriaceae (CRE). This underscores the reality that engineering redesigns, rigorous quality systems, and broader adoption of single-use technologies are not optional, but essential, to move toward eliminating this preventable source of healthcare-associated infection [5].

Reprocessing of the duodenoscopes remains a cornerstone of infection prevention in gastrointestinal endoscopy, given the well-documented risk of patient-to-patient transmission of multidrug-resistant organisms through inadequately reprocessed instruments [6,7]. The complex design of the duodenoscopes—particularly the presence of the elevator mechanism and associated narrow recesses—creates significant challenges for complete decontamination and high-level disinfection (HLD), even when protocols are correctly followed [8,9,10].

Current international guidelines advocate a structured, multistep reprocessing workflow encompassing pre-cleaning, manual cleaning, high-level disinfection, drying, and continuous quality assurance [9,10]. Pre-cleaning should occur immediately after the procedure, with detergent solution aspirated through all channels to prevent drying of organic material and facilitate later cleaning [10,11]. Manual cleaning involves leak testing, disassembly of detachable components, and meticulous brushing and flushing of all internal channels and the elevator recess using enzymatic detergent, followed by thorough rinsing with clean water [9,12]. Subsequently, the duodenoscope undergoes HLD—either within an automated flexible endoscope reprocessor or by validated manual immersion—using chemical agents such as glutaraldehyde, ortho-phthalaldehyde, or peracetic acid, with strict adherence to manufacturer-specified contact times and temperatures [9,10,11]. However, it has been previously shown that HLD cycles and routine instrument/brush passage can produce cumulative surface defects (scratches, pits, residue) and channel roughening, creating niches for biofilm and microbial persistence despite guideline-concordant reprocessing [12,13].

Following HLD, channels must be flushed with sterile or bacteria-free water and purged with forced air. Active drying—often preceded by an alcohol flush—is indispensable, as residual moisture promotes microbial proliferation and biofilm formation [7,10]. The processed duodenoscope should then be stored in a ventilated drying cabinet with continuous filtered airflow to prevent recontamination [10,12]. Finally, guidelines emphasize quality assurance, surveillance, and documentation; reprocessing records should be maintained, and regular microbiological culturing—particularly of the elevator mechanism and distal tip—should be performed as part of ongoing surveillance [9,10,13].

Despite adherence to best practices, duodenoscope-associated infections continue to be reported, underscoring inherent limitations of current designs and reprocessing technologies [8,13]. Consequently, expert societies and regulatory agencies advocate supplementary mitigation strategies—such as double HLD cycles, ethylene oxide sterilization, or “culture-and-hold” protocols—and encourage progressive transition to the duodenoscopes with disposable distal caps or fully single-use designs [8,10,14,15].

Our earlier studies proved the effects of sterilisation cycles on the duodenoscope, emphasising changes to the coatings and working channel polymers due to intensive usage, mostly to the proximal part [16]. Additionally, our previous studies emphasized that there are more friendly alternatives to the duodenoscope reprocessing than those frequently used [17,18]. In the same framework, we approached calcium peroxide as a metastable compound that has been widely investigated for environmental and biomedical applications. Previously, calcium peroxide has been employed in wastewater treatment [19], and sludge treatment [20], for the removal of organic pollutants, due to its strong oxidative reactivity. In addition, it exhibits remarkable antimicrobial activity [21], and has been explored as an oxygen-releasing material in tissue engineering [22,23], highlighting its versatility in biomedical applications. Recently, we reported the synthesis of the CaO_2_−Alg NPs [24], with a high specific surface area. Importantly, these nanoparticles possess a relatively low oxidizing potential, allowing them to retain their antimicrobial efficacy while minimizing damage to disinfected surfaces.

When used as part of the HLD process, they would gradually release reactive oxygen species, primarily hydrogen peroxide and hydroxyl radicals, providing sustained antimicrobial activity while minimizing surface corrosion and toxic residue formation. The alginate matrix enhances stability and controlled release, allowing efficient biofilm disruption within narrow luminal channels. Preliminary experimental data suggest that the CaO_2_–Alg formulations could achieve broad-spectrum bactericidal and sporicidal efficacy comparable to peracetic acid or glutaraldehyde, but under milder conditions and with reduced environmental impact on medical devices [24].

In this context, the aim of our study was, for the first time, to evaluate an alternative disinfectant based on the CaO_2_–Alg NPs to improve the duodenoscope reprocessing and reduce the incidence of nosocomial infections associated with the use of this medical device. Additionally, we assessed the impact of NP treatment on the structure and properties of the polymer external coating after intensive use.

## 2. Materials and Methods

### 2.1. Duodenoscope Samples

**Figure 1:**
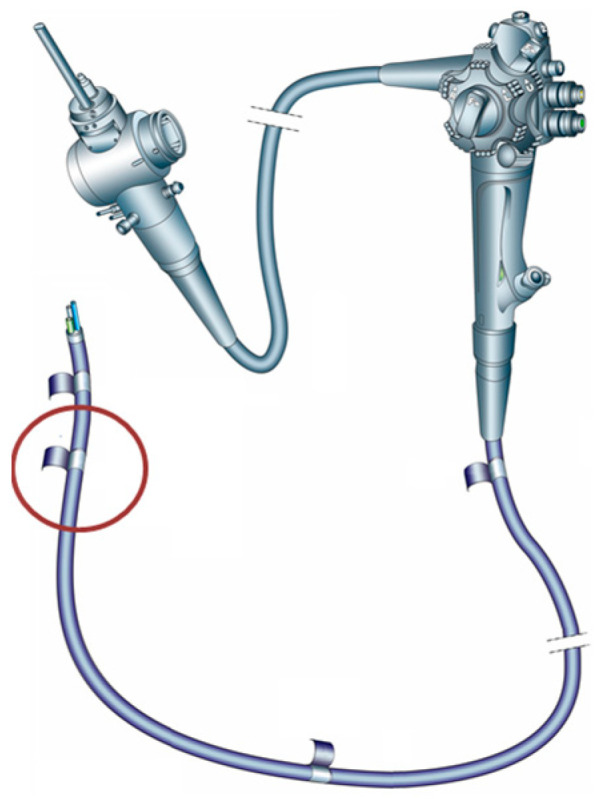
Duodenoscope scheme with the sampling site marked in red.

Samples were collected from a reference duodenoscope, TJF-160F, Olympus Corporation, Tokyo, Japan, previously used in a gastroenterology center for approximately 500 endoscopic retrograde cholangiopancreatography (ERCP). The duodenoscope was quarantined for six months and was manually reprocessed according to the manufacturer’s revised instructions. HLD was performed using a peracetic acid solution. Subsequently, cultures were collected from the external surfaces of the elevator, working channels, water, and air. The culturing process involved a combination of channel flushing, channel brushing, and elevator swabbing, which was subsequently followed by inoculation in a liquid medium of tryptic soy broth at 37 °C for 48 h. All cultures were negative, which allowed the duodenoscope to be disassembled, and approximately 1 cm^2^ study samples of the outer coating polymers were taken from the area 10–20 cm from the distal end (as presented in Figure 1). The disassembly process was performed in a microbiologically controlled environment using a Class II microbiological safety cabinet (Bioquell, Andover, UK).

### 2.2. The CaO_2_–Alg NPs Synthesis

The CaO_2_–Alg NPs were prepared by chemical precipitation with H_2_O_2_ in the presence of sodium alginate as surfactant, following our previously reported protocol without any modifications [24]. The formation and physico-chemical characterization of synthesized NPs were investigated by X-ray diffraction, Fourier transform infrared spectroscopy, electronic microscopy, dynamic light scattering, electron spin resonance, and thermal analysis, as described in our earlier work.

### 2.3. Biological Investigation

For the microbiological assays there were four reference bacterial strains used, represented as follows: *Staphylococcus aureus* ATCC25923 (*S. aureus*), *Escherichia coli* ATCC25922 (*E. coli*), *Enterococcus faecalis* ATCC29212 (*E. faecalis*), and *Klebsiella pneumoniae* ATCC10031 (*K. pneumoniae*).

#### 2.3.1. Evaluation of the Disinfectant Properties of the CaO_2_–Alg NPs Under Biomimetic Conditions

The study samples were contaminated with bacterial suspensions in biomimetic medium and subsequently treated with the CaO_2_–Alg NPs at successive time intervals. The control samples were isolated in sterile plates. The experiment was performed in three replicates at each sampling location. To obtain biomimetic conditions, 10 mL of bacterial suspension standardized at 10^8^ CFU/mL in ultrapure sterilized water was mixed with 90 mL of standardized intestinal biomimetic fluid FeSSIF (Fed State Simulated Intestinal Fluid) (Biorelevant, London, UK), which replicates the environment after a high-fat meal, containing an increase in bile salts and lipids, to simulate a physiologically contaminated environment. The mixture was continuously maintained at a temperature of 37 °C. The duodenoscope samples were immersed in the bacterial solution under biomimetic conditions for 15 min at a temperature of 37 °C, with continuous mixing.

After this interval, the samples were taken from the medium and left on a sterile surface for 1 min to dry and subsequently put in contact with the CaO_2_–Alg NPs suspension in successive stages for 5, 10, 15, 20, and 30 min, respectively, with periodic shaking every 5 min. In parallel, the control samples were immersed in sterile distilled water. After treatment with the nanoparticles or distilled water, respectively, the samples were left to dry on a sterile surface for 1 min. After drying, they were transferred to containers containing 30 mL of sterile tryptone-soy broth medium, sealed, and incubated at 37 °C for 72 h. The presence of turbidity following incubation suggested bacterial growth, indicating the presence of viable microorganisms in the samples post-disinfection. Conversely, the absence of turbidity after incubation demonstrated a lack of viable microorganisms, confirming the disinfectant efficacy of the CaO_2_–Alg NPs for the specified contact duration. All incubated bottles, regardless of the appearance of the media, were subcultured on appropriate solid media to confirm the absence of viable bacteria. Negative subcultures and the lack of turbidity (clear medium) were considered indicative of HLD. All experiments were conducted in a Class II microbiological safety cabinet (Bioquell, Andover, UK) to prevent contamination with extraneous microbial species.

#### 2.3.2. Evaluation of the Compatibility of the CaO_2_−Alg NPs with the Polymeric Coating of the Duodenoscope

To evaluate the effect of the repeated CaO_2_–Alg NP treatments on the duodenoscope polymer structure, a challenge test was conducted. Contaminated device samples were immersed in the CaO_2_–Alg NP suspensions for 30 min daily over a 45-day period. Control samples were treated under identical conditions, using distilled water instead of the CaO_2_–Alg NPs. All samples (both treated and untreated) were analyzed by scanning electron microscopy (SEM), atomic force microscopy (AFM), and energy-dispersive X-ray spectroscopy (EDAX).

Fourier Transform Infrared Spectroscopy (FT-IR)

FT-IR spectra were obtained using a Bruker Vertex 70 FT-IR spectrometer (Billerica, MA, USA), conducted at room temperature with a resolution of 2 cm^−1^ in the range of 500–4000 cm^−1^.

Differential Scanning Calorimetry (DSC)

DSC measurements were recorded on a DSC 200 F3 Maia device (Netzsch–Gerätebau GmbH, Selb, Germany). 8 mg of each sample was heated in aluminum crucibles sealed shut with pierced lids. Nitrogen atmosphere was used (flow rate of 50 mL min^−1^) and a heating rate of 10 °C min^−1^ was applied. Calibration was conducted with standard indium.

Atomic Force Microscopy (AFM)

Images from the study samples collected from the surface of the duodenoscope were recorded in air, in tapping mode using an NTEGRA Spectra (NT-MDT, Zelenograd, Russia) instrument with a 3.1–37.6 N/m force constant cantilever of silicon nitride cantilevers (NSC10, NT-MDT, Moscow, Russia).

Scanning Electron Microscopy (SEM)

The surface morphology of the study samples was observed using a Quanta200 scanning electron microscope (FEI Company, Hillsboro, OR, USA), working in a low vacuum mode, at 20 kV with an LFD detector. To obtain the elemental information, EDX analysis using a silicon drift detector was performed on both controls and samples.

#### 2.3.3. Evaluation of Microbial Adhesion on the Duodenoscope Surface Following Prolonged Contact with Metal Oxide Nanoparticles

The anti-adhesion properties of polymer samples after treatment with the CaO_2_–Alg NPs were investigated in accordance with the Japanese Industrial Standard JIS Z2801:2000 [25]. The JIS Z 2801:2000 method is a standardized test to quantitatively measure the antibacterial activity of antimicrobial surfaces, such as plastics, ceramics, and metals, by inoculating them with bacteria and incubating for 24 h. After incubation, the number of surviving bacteria is counted to calculate the log reduction, which indicates the surface’s efficacy in inhibiting or killing microorganisms. The same bacterial strains were activated in liquid culture medium by incubation for 24 h at 37 °C. The preparation of the tested surfaces involved placing each sample in a sterile Petri dish. The bacterial inoculum was adjusted to standard values of 0.5 McFarland with dilutions to obtain a bacterial concentration of 2.4 × 10^5^ CFU/mL. Subsequently, 0.1 mL of the obtained inoculum was placed on the studied samples and these were subsequently incubated for 24 h at 37 °C. After incubation, the samples were rinsed repeatedly with sterile ultrapure water, and the resulting suspension underwent binary dilutions, which were cultured on PCA (plate count agar) medium and incubated for 24 h at 37 °C, and then the number of resulting colonies was recorded. The interpretation of the results was conducted according to the specifications of JIS Z2801:2000 Tokyo, Japan.

## 3. Results and Discussion

### 3.1. Evaluation of the Disinfectant Properties of the CaO_2_–Alg NPs Under Biomimetic Conditions

The impact of repeated exposure of polymer resins on the duodenoscope surface to the CaO_2_–Alg NPs was assessed using a previously presented case-control experimental study. The results of the antimicrobial activity of the CaO_2_–Alg NPs are presented in Table 1. Thus, after 5, 10, and 15 min of exposure, all four strains could still be identified on control cultures. After 20 min of treatment with the CaO_2_–Alg NPs, both liquid and solid cultures showed negative results, consistent with the absence of bacterial growth and, therefore, with HLD. The results were the same after 30 min of incubation. The total absence of all four bacterial strains after 20 min of sample exposure proves the high-grade disinfectant activity on polymer samples.

The CaO_2_ NPs have been employed extensively in different fields for the treatment of bacterial contamination, because they provide oxygen (O_2_) and hydrogen peroxide (H_2_O_2_) to the treatment site [21,26,27,28,29]. In dry settings, the CaO_2_ is quite stable, but in an aqueous environment, it slowly decomposes. According to the literature, the CaO_2_ has also been utilized to cure cancer because it decomposes to H_2_O_2_ more quickly in an acidic environment [30,31,32,33]. The bacterial infection site pH is substantially lower than that of the surrounding tissues, and it is very similar to the microenvironment of a tumour; it has been proven that the CaO_2_ NPs’ rapid release of the H_2_O_2_ may destroy the bacterial cells [34]. Previously, we have proved that the release of hydrogen peroxide increases significantly at low pH compared to neutral pH [24].

These processes could provide an advantage for the use of the CO_2_ as a disinfectant, because in the immediate vicinity of the acidic bacterial environment, there is an increase in the concentration of the Ca^2+^ and the H_2_O_2_, while in the rest of the dispersion medium, the oxidative and calcium ion levels are lower. H_2_O_2_–induced oxidative stress targets important bacterial components such as lipids, proteins, and nucleic acids, leading to cellular dysfunction and then cell death [35,36,37,38,39]. The calcium ions may also contribute to the antibacterial efficiency by disrupting cell membranes [21,40,41]. Moreover, these NP may inhibit the proliferation of bacterial biofilms, primarily by causing oxidative damage to the extracellular polymeric substances (EPS) from the biofilm structure [35,36,37]. Therefore, the CaO_2_ NPs may be excellent options for the duodenoscope reprocessing.

### 3.2. Evaluation of the Compatibility of the CaO_2_–Alg NPs with the Polymeric Duodenoscope Coating

Elemental composition analysis studies of the duodenoscope samples were carried out using the X-ray spectroscopy technique (EDAX), which highlighted the presence of the atoms that form the material covering the duodenoscope, namely: C, N, O, Na, Mg, P, S, and K, in different atomic percentage ratios, as presented in Table 2. Compared to the duodenoscope control sample, for the sample treated with the CaO_2_–Alg NPs, a slight increase in the mass percentages of C (from 66.59% in the control sample to 68.72% for the treated sample) and O (from 18.65% in the control sample to 21.7% for the treated sample) were observed, as well as the appearance of the Ca atom (0.79 atomic%), which is due to the presence of traces of the CaO_2_–Alg NPs on the surface of the material as a result of the material disinfection. The elemental composition of the samples, which contains: C, N, O, Na, Mg, Al, P, S, and K, is similar to the one revealed by our previous study [17]; the difference between the percentages is caused by the fact that this study is based on samples from a single area of the duodenoscope.

These traces of NPs are not significant because they can be easily removed by a final washing step of the disinfection process. Additionally, as previously studied by our group, in case of remanence on the duodenoscope surface after completing the reprocessing process, these NPs are biocompatible up to a maximum concentration of 25 μg/mL for the two tested cellular lines taken into study, namely, Normal Human Dermal Fibroblast (NHDF) and Human Gingival Fibroblast (HGF) [24].

**Figure 2:**
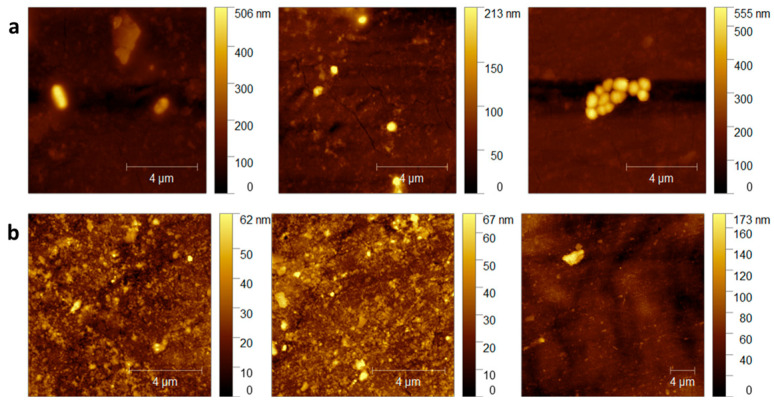
AFM morphological evaluation of control (**a**), and disinfected samples (**b**).

The AFM morphological analysis of the duodenoscope samples shown in (Figure 2) did not reveal any evident new lesions of the polymeric surface of the duodenoscope following exposure to the CaO_2_–Alg NPs when compared with the controls. These conclusions correlated with the previously presented chemical analysis of the coating samples, which may lead to the conclusion that the intensive use of the NPs does not bring additional damage to the external duodenoscope coating. However, in comparison, bacterial colonies and biofilm formation can be observed on the control samples shown in (Figure 2a), whereas these features are absent on the samples treated with the CaO_2_–Alg NPs (Figure 2b). On the treated and disinfected surfaces, traces of nanoparticles were still visible but could be easily removed during the final washing step of reprocessing. The morphology of the untreated samples was consistent with the topographic images obtained in our previous study, which revealed an inhomogeneous surface and the presence of microcracks resulting from the intensive use of the duodenoscope, leading over time to polymer degradation [16].

**Figure 3:**
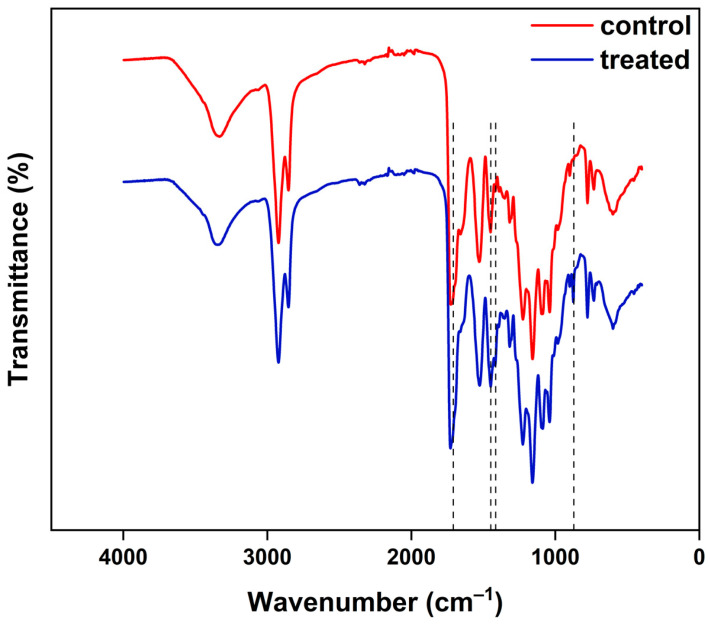
ATR-FT-IR spectra for control (red) and treated (blue) duodenoscope samples.

Structural characterization of the material used to cover the duodenoscope was evaluated using the FT-IR technique. For the analysis, two material samples were collected from the same area of the duodenoscope, one control sample and one sample treated with the CaO_2_–Alg NPs for 45 days. From the FT-IR spectra analysis of the two samples shown in (Figure 3), it may be observed that the absorption bands do not show major changes following treatment with the CaO_2_–Alg NPs disinfectant. The structural characterization of the control and treated samples is in accordance with the characterization made by our group in a previous study that emphasized structural changes of the external polymer due to intensive usage of the duodenoscope [16].

Both spectra display absorption bands at 2900 and 2853 cm^−1^, respectively, characteristic of the stretching vibrations of the CH and CH_2_ groups, the absorption bands present in the 1760–1600 cm^−1^ region are characteristic of the vibrations of the C=O and C=N groups, and those in the 1040-1200 cm^−1^ region are generally associated with the vibrations of the C–O, C–O–C, C–N groups. Compared to the control sample, the treated material sample shows traces of the CaO_2_–Alg NPs that can be observed in the FT-IR spectrum of the treated sample through slightly more intense vibrational bands at 1611 and 1418 cm^−1^, which were attributed to the symmetric and antisymmetric stretching vibrations of the COO^−^ group salt [35], through the intensification of the band at 1449 cm^−1^ attributed to the O–Ca–O deformation vibration as well as the appearance of the absorption band at 876 cm^−1^ corresponding to the stretching vibration of the peroxide group, O–O [24].

The present findings are consistent with our previous reports, which indicated that the CaO_2_–Alg NPs generate a moderately oxidative environment under neutral conditions, as suggested by the electron spin resonance (ESR) analysis of free radicals produced by the hydrogen peroxide released [24]. This suggests that by moderating the oxidizing capacity while maintaining effective disinfectant properties, these nanoparticles offer an advantage by minimizing surface damage to the duodenoscope during the disinfection process.

**Figure 4:**
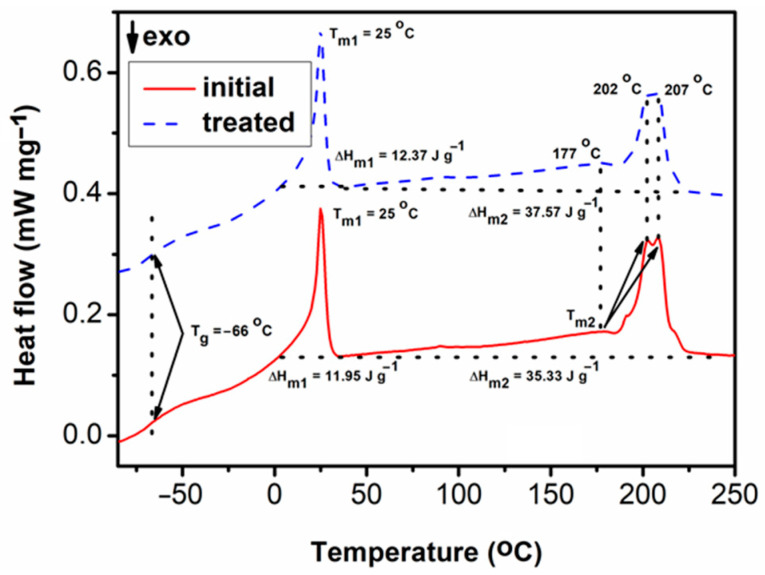
Representative DSC curves of untreated (blue) and CaO_2_–Alg NP—treated (red) duodenoscope samples.

Figure 4 shows the second DSC heating curves of the studied materials. It may be observed that the initial and treated samples show no difference in the glass transition temperature (Tg = −66 °C). The same is in the case of the two melting profile temperatures [16], the one at a lower temperature (T_m1_ = 25 °C) and the multiple peaked one at higher temperatures (T_m2_ = 177 °C, 202 °C and 207 °C). By analysing the DSC data, it may also be observed that there is only a slight increase in the melting enthalpy (ΔH_m_) values. ΔH_m1_ increases from 11.95 J g^−1^ for the initial sample to 12.37 J g^−1^ for the treated sample, and ΔH_m2_ increases from 35.33 J g^−1^ for the initial sample to 37.57 J g^−1^ for the treated sample. All data indicate that the treatment process does not induce noteworthy modifications in the thermal behaviour of the material.

### 3.3. Evaluation of Microbial Adhesion on the Duodenoscope Surface Following Prolonged Contact with the CaO_2_–Alg NPs

**Figure 5:**
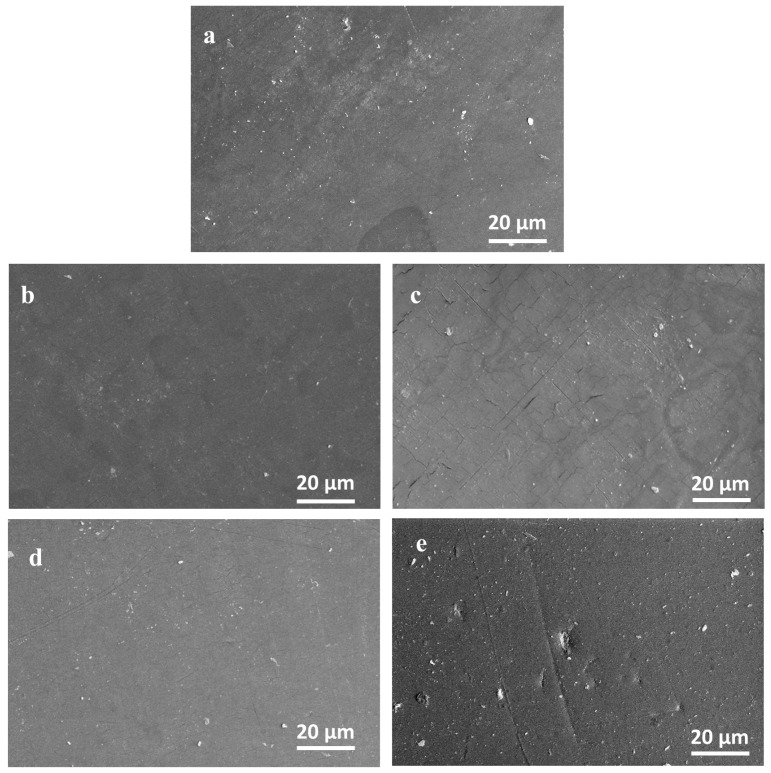
Scanning electron microscopy (SEM) evaluation of duodenoscope samples: control (**a**) infected with *S. aureus,* (**b**) *E. coli,* (**c**) *E. faecalis,* (**d**) *K. pneumoniae,* (**e**) and disinfected with CaO_2_–Alg NPs.

**Figure 6:**
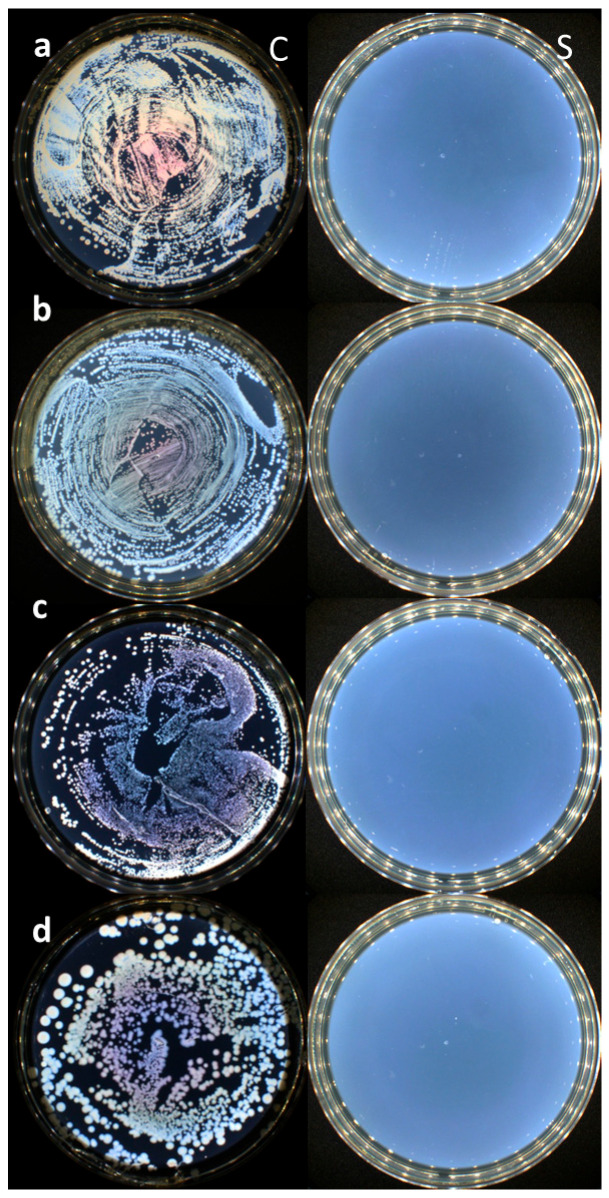
Antibacterial efficiency of CaO_2_−Alg NPs (S) compared with the controls (C) against the following: (**a**) *S. aureus*, (**b**) *E. coli*, (**c**) *E. faecalis*, and (**d**) *K. pneumoniae*.

All the duodenoscope samples studied in contact with the CaO_2_–Alg preserved their anti-adherence properties according to the Japanese Industrial Standard JIS Z2801:2000. Thus, no adhesion and bacterial growth processes were evidenced for the Gram-positive (*S. aureus*, *E. faecalis*) and Gram-negative (*E. coli*, *K. pneumoniae*) bacterial strains tested as revealed by the SEM analysis of the treated samples and controls (see Figure 5). The duodenoscope surface seems to present the same erosion and deterioration induced by the intensive usage and repeated reprocessing as presented by our team in a previous study [16]. Moreover, no bacterial incubation was identified in the Petri plates after 24 h of incubation at 37 °C (Figure 6), which proves the disinfectant properties of the CaO_2_–Alg NPs.

These results are also in accordance with our previously made studies on an in-use duodenoscope [16], proving that the material is preserving its antibacterial properties after the prolonged and intensive utilization of the new proposed disinfectant represented by the CaO_2_–Alg NPs.

### 3.4. Evaluation of the Economic Burden and Subsequent Challenges of Single-Use Versus Current Multiple-Use Reprocessible Duodenoscopes

Although single-use duodenoscopes offer the theoretical advantage of eliminating cross-infection from reuse, their adoption in general practice has been slow and remains limited by several practical, environmental, and economic constraints. Recent analyses suggest that single-use duodenoscopes incur a higher per-procedure cost compared to well-maintained reusable systems, although the balance may shift in certain scenarios (e.g., low-volume centers or high-infection-risk). A systematic review of economic evaluations concluded that per-procedure cost has generally been higher using single-use endoscopes (versus reusable), although a subset of cost-utility analyses showed single-use designs could become cost-effective under favourable assumptions, especially regarding the high infection costs [42]. A recent European Societies’ position statement emphasizes concerns about the environmental and waste burden of routine single-use devices and suggests against their indiscriminate use, recommending instead a case-by-case assessment [43,44,45]. Meanwhile, when analysing the economic burden and cost trade-offs of all enhanced reprocessing practices currently recommended, one could easily conclude that widespread implementation of single-use duodenoscopes would impose substantial capital and operational costs on health care systems accustomed to high volumes of ERCP and reuse workflows [3,46,47]. Another analysis calculated break-even pricing for single-use devices; in the Dutch setting, single-use scopes would need to be priced between €140 and €250 to rival reusable scopes when used selectively in high–risk patients [48]. Even in such selected scenarios, their numbers are far from the current economic reality.

Thus, despite recognized infection risks from reusable scopes, the balance of sustainability, cost, infrastructure, and waste management continues to hinder the broad deployment of single-use duodenoscopes in favour of reprocessing multiple-use duodenoscopes. Hence, there is still a constant need for optimizing reprocessing cycles in both efficacy and long-term sustainability, both pleading for potential alternative environmental and scope-friendly measures. However, in low-volume or high-risk settings, single-use devices may approach financial viability under favourable pricing, reimbursement, or infection-cost assumptions.

## 4. Conclusions

Although this is clearly a preliminary study, due to the limited evaluation of the CaO_2_–Alg NP disinfectant properties and their effects on different regions of the duodenoscope with varying materials, the present research provides novel insights and demonstrates the potential utility of these nanoparticles as an alternative in the duodenoscope reprocessing. The goal is to optimize bacterial and biofilm removal while minimizing polymer resin degradation during reprocessing. Although still at an exploratory stage, integrating such biomaterials into reprocessing workflows may offer a pathway to reduce contamination rates and mitigate infection risk, thereby enhancing patient safety in endoscopic practice.

## Figures and Tables

**Table 1:** Evaluation of the antimicrobial activity of CaO_2_–Alg NPs at different time intervals.

Tested Strain	5 min	10 min	15 min	20 min	30 min
*E. coli*	+	+	+	−	−
*K. pneumoniae*	+	+	+	−	−
*E. faecalis*	+	+	+	−	−
*S. aureus*	+	+	+	−	−

Note: + indicates the presence of turbidity in the culture media (microbial growth); − indicates no turbidity (confirmed by complete absence of growth on subcultures, that is, HLD).

**Table 2:** Mass and atomic ratios obtained by the EDAX technique following the analysis of the controls and CaO_2_–Alg NPs treated samples.

Element	Control		Treated Sample	
	Weight %	Atomic %	Weight %	Atomic %
C	59.14	66.59	59.65	68.72
N	11.98	11.56	5.40	5.33
O	22.07	18.65	25.09	21.70
Na	1.47	0.86	0.57	0.34
Mg	0.50	0.28	0.40	0.23
P	2.68	1.17	4.29	1.92
S	1.71	0.72	1.28	0.55
K	0.47	0.16	0.63	0.22
Ca	-	-	2.29	0.79

## Data Availability

The original contributions presented in this study are included in the article. Further inquiries can be directed to the corresponding authors.
